# Hybrid Protective Sentinel System: A Combined Technique for Safe and Controlled Open‐Wedge High Tibial Osteotomy

**DOI:** 10.1002/atn2.70281

**Published:** 2026-09-25

**Authors:** Ashraf Elazab, Mohamed Darwish, Ibrahim Mandour, Mohamed Gad, Bassem Abdelmotalleb Elfeky, Hesham Gawish

**Affiliations:** ^1^ Orthopedic Surgery Department Mansoura National University and Mansoura International Hospital Mansoura Egypt; ^2^ Minstry of Health Mansoura Egypt; ^3^ Department of Orthopedic Surgery Faculty of Medicine Al‐Azhar University Damietta Egypt; ^4^ Department of Orthopedic Surgery and Traumatology Faculty of Medicine Kafr El‐Sheik University Kafr El‐Sheikh Egypt

## Abstract

Open‐wedge high tibial osteotomy is an effective joint‐preserving surgery for medial compartment osteoarthritis but remains associated with 3 major intraoperative hazards: posterior neurovascular injury, lateral hinge fracture, and unintended slope modification. The present technical note describes a hybrid method that integrates the protective cutting system and sentinel wires technique to achieve maximal procedural safety during open‐wedge high tibial osteotomy.

## Graphical abstract

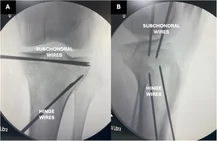

**VIDEO 1:** A 6‐cm longitudinal incision is made just medial to the tibial tuberosity, extending proximally to the joint line. Subcutaneous dissection exposes the sartorial fascia, which is incised in line with its fibers, and the superficial medial collateral ligament is elevated from its tibial insertion to expose the anteromedial cortex. Before performing the osteotomy, a sentinel wire construct is inserted to protect the lateral hinge and control saw depth. Two hinge K‐wires are introduced from the medial cortex toward the lateral hinge, intersecting the planned osteotomy plane approximately 10 mm medial to the lateral cortex to limit saw depth and prevent type 2 hinge fractures while avoiding anterior saw escape. A subchondral K‐wire is then placed just beneath the joint line, parallel to the articular surface, to prevent superior type 3 hinge fractures. The protective cutting system (Tradimedics, Seongnam, Republic of Korea) is subsequently introduced along the posteromedial tibial cortex to shield posterior neurovascular structures. Under fluoroscopic guidance, the protector tip is positioned at the planned hinge point, and the cutting block is aligned with the native tibial slope and fixed to the medial cortex. A horizontal osteotomy is performed through the guided slot toward the protector tip, ensuring controlled posterior cortex cutting, followed by a vertical cut at approximately 110° to create a biplanar configuration while preserving at least 10 mm of the lateral cortex. The osteotomy is gradually opened using osteotomes and lamina spreaders with the sentinel wires in place. Final fixation is achieved using a medial TomoFix locking plate, with bone grafting added if the opening exceeds 10 mm. Video content can be viewed at https://doi.org/10.1002/atn2.70281.

Open‐wedge high tibial osteotomy (OWHTO) is a widely accepted treatment for medial compartment osteoarthritis in young and active patients with varus malalignment.^1^, ^2^, ^3^ Despite its proven clinical benefits, the procedure remains technically demanding, with intraoperative complications that can compromise both safety and outcome. The most frequent technical issues are injury to the posterior neurovascular structures, tibial hinge fractures, and the uncontrolled changes in the posterior tibial slope.^4^, ^5^, ^6^, ^7^

To reduce these risks, several authors have developed adjunctive safety techniques. In 2016, Lee et al. introduced the protective cutting system (PCS, Tradimedics, Seongnam, Republic of Korea), a curved metallic guide that isolates the posterior neurovascular bundle and constrains the saw blade within a preformed slot.^8^ This system allowed controlled osteotomy along the natural tibial slope, complete posterior wall cutting, and protection of vital posterior structures. However, although PCS effectively prevents neurovascular injuries and saw misdirection, it does not safeguard the lateral cortical hinge from fracture. In contrast, Gawish et al. in 2025 described the sentinel wires technique, which enhances lateral hinge stability by inserting 3 K‐wires—the osteotomy, hinge, and subchondral wires—forming a “C‐shaped” construct that reinforces the lateral hinge and prevents Takeuchi type 2 and 3 fractures.^9^

Given the complementary safety mechanisms of these 2 techniques, this article describes a hybrid protective sentinel system (HPSS) that merges the advantages of both techniques. The system uses the PCS to guide and restrict saw motion, ensuring posterior safety, while simultaneously employing sentinel wires to protect the hinge and maintain osteotomy integrity. This hybrid method aims to provide full‐spectrum protection during the tibial cut, guarding posterior neurovascular structures and preventing hinge fractures while controlling the posterior tibial slope through a reproducible, safe technique.

## SURGICAL TECHNIQUE

This technical note describes the technique of HPSS for OWHTO (Video 1). The details are summarized in Tables 1 and 2.

**TABLE 1:** Advantages and Disadvantages of the Technique

**Advantages**	**Disadvantages**	**Our Modification/Note**
Enhanced hinge protection through the addition of 2 sentinel subchondral and hinge wires	Introduces additional surgical steps, including insertion of multiple K‐wires and assembly of the PCS, which may increase operative time during the learning phase	Use of 2 H‐wires instead of 1 provides superior hinge protection
Facilitates accurate reproduction of the native posterior tibial slope using the cutting block component of the PCS	Requires availability of the PCS instrumentation, potentially limiting use in centers without access to the system	Combines PCS guidance with hinge protection for controlled slope reproduction
Allows complete posterior cortex cutting with no vascular risk	Improper placement of sentinel wires may interfere with saw trajectory or plate positioning, necessitating careful planning	Hybrid integration ensures posterior safety while protecting the hinge
Provides clear mechanical cutting boundaries, improving osteotomy precision and reproducibility	Does not entirely eliminate the risk of lateral hinge fracture, particularly in osteoporotic bone or with excessive correction angles	Mechanical stop with 2 H‐wires enhances reproducibility and hinge preservation
Does not require complex or expensive navigation systems	Inserting multiple wires may be technically demanding in narrow tibial segments or small female knees	Technical challenge acknowledged, but safety gain outweighs difficulty

H‐wires, hinge wires; PCS, protective cutting system.

**TABLE 2:** Pearls and Pitfalls of the Technique

**Pearls**	**Pitfalls**
Accurate preoperative planning of the hinge location is critical, with the protector tip and hinge wires positioned 10 to 15 mm medial to the lateral cortex at the level of the fibular head	Inadequate posterior soft‐tissue release may prevent proper seating of the PCS
Maintaining constant bone contact while introducing the PCS releaser ensures safe creation of the posterior corridor and minimizes soft‐tissue injury	Misalignment of the cutting block may result in unintended posterior tibial slope changes
Using 2 hinge wires instead of a single wire effectively prevents anterior saw escape and enhances hinge stiffness	Hinge wires placed too laterally increase fracture risk; too medial placement may limit correction
Alignment of the PCS cutting block with the native posterior tibial slope using a true lateral fluoroscopic view is essential to avoid unintended slope alteration	Incomplete posterior cortex cutting before wedge opening increases hinge propagation risk
Gradual and symmetric wedge opening under fluoroscopic control reduces stress concentration at the hinge and complements the protective role of the sentinel wires	Premature removal of sentinel wires before fixation negates their protective role
	Improper MCL dissection may lead to MCL injury and medial instability with the use of the bulky PCS

MCL, medial collateral ligament; PCS, protective cutting system.

### Patient Positioning

The patient is placed in the supine position on a radiolucent operating table. A well‐padded high‐thigh tourniquet is applied, and a leg support is used to maintain 90° of knee flexion.

### Approach and Exposure

A 6‐cm longitudinal incision is made just medial to the tibial tuberosity, extending proximally toward the joint line. Subcutaneous dissection exposes the sartorial fascia and the superficial medial collateral ligament. The pes anserinus is incised in line with its fibers, and the medial collateral ligament is carefully elevated from its tibial insertion to expose the anteromedial cortex.

A blunt curved Cobb elevator is introduced posteriorly to identify and free the posteromedial tibial surface. This dissection is essential to create the plane for the PCS (Tradimedics, Seongnam, Republic of Korea) insertion, ensuring a safe corridor to the posterior cortex while retracting soft tissues posteriorly.

### Step 1: Insertion of Sentinel Wires

**FIGURE 1:**
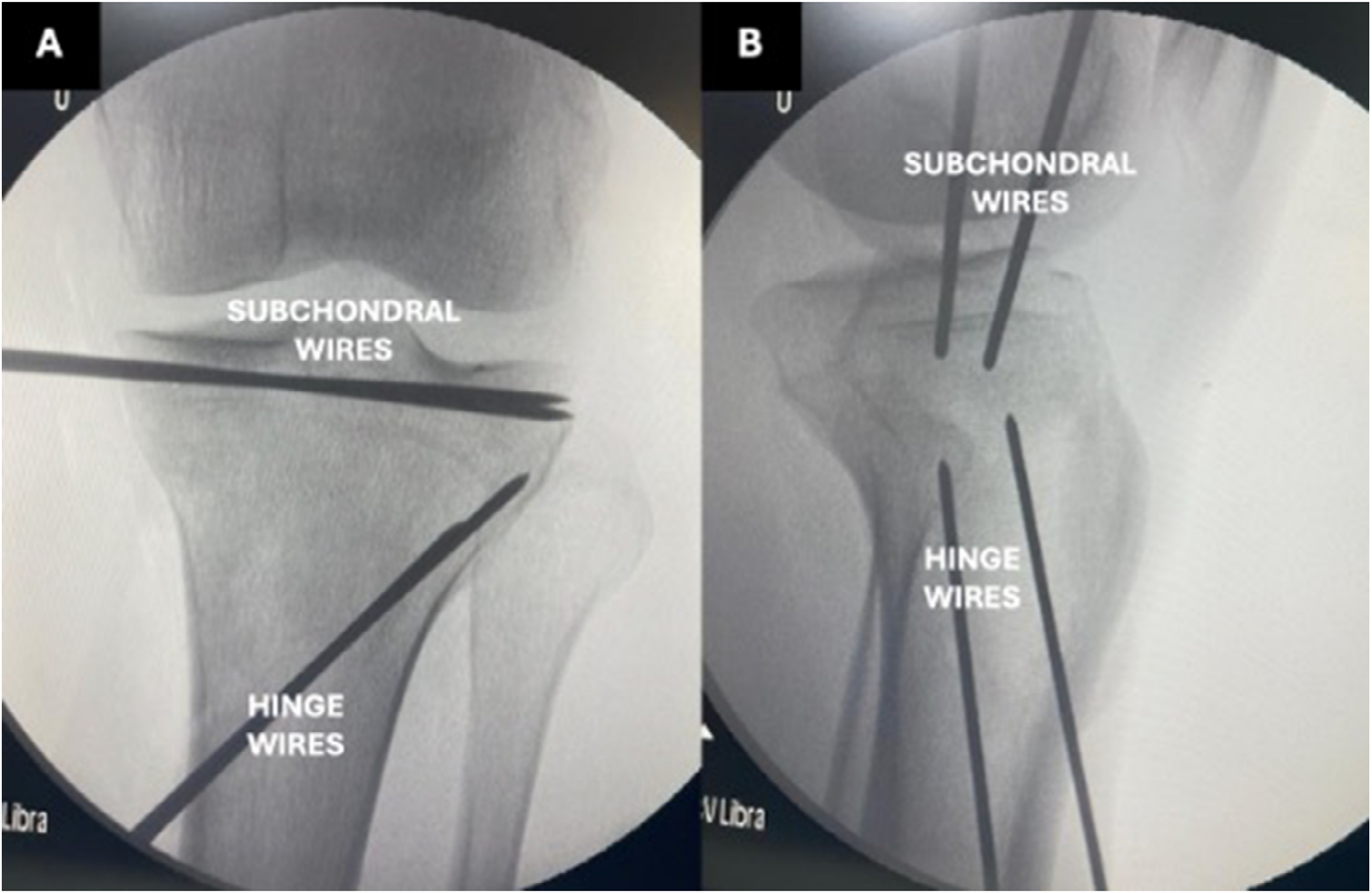
A fluoroscopic view of left proximal tibia showing the orientation of the subchondral and hinge wires. (A) Anteroposterior view. (B) Lateral view.

**FIGURE 2:**
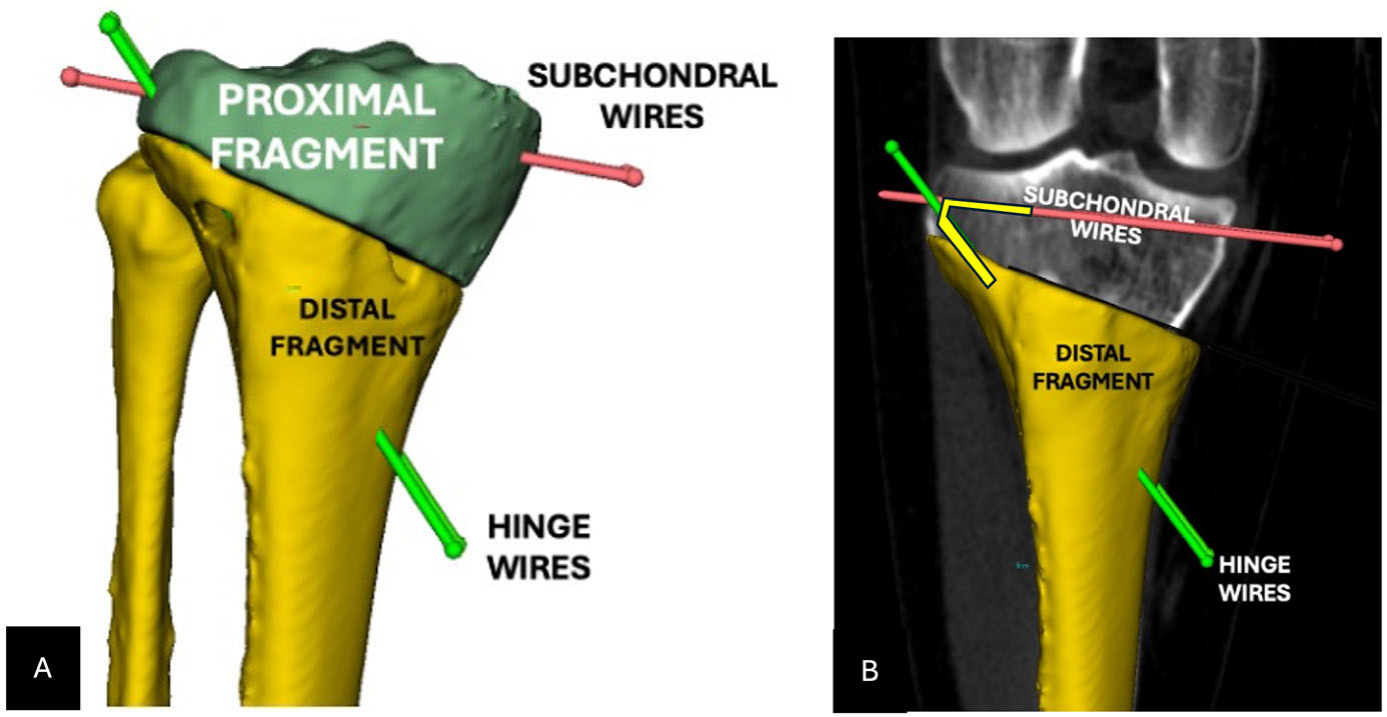
(A) A 3D illustration of right proximal tibia showing the orientation of the subchondral and hinge wires. (B) A 3D illustration over a coronal CT cut of right proximal tibia showing the orientation of the subchondral and hinge wires in C‐shaped manner. (3D, 3‐dimensional; CT, computed tomography.)

Before initiating the osteotomy, 3 sentinel K‐wires are inserted to reinforce the lateral hinge and control saw depth (Figures 1 and 2).

First is the insertion of hinge wires: 2 K‐wires (2.0 mm) are introduced from the medial cortex toward the lateral hinge, intersecting the planned osteotomy plane approximately 10 mm medial to the lateral cortex. These wires limit saw depth and resist propagation of type 2 hinge fractures. Moreover, in the present technique, 2 K‐wires were deliberately used instead of a single K‐wire, as described in the original sentinel wires technique by Gawish et al., to prevent anterior escape of the saw blade toward the anterior cortex.

Second is insertion of the subchondral wires: 2 subchondral wires (2.0 mm) are placed just beneath the joint line, parallel to the articular surface, to prevent superior (type 3) hinge fractures through the lateral plateau. The combined arrangement of these wires forms a C‐shaped sentinel construct, enhancing lateral hinge stiffness and defining the safe cutting boundaries for the saw (Figure 2b).^9^

### Step 2: Insertion of the PCS

The PCS (Tradimedics, Seongnam, Republic of Korea) consists of a releaser and a protective cutting complex comprising a curved protector and cutting block.^8^

**FIGURE 3:**
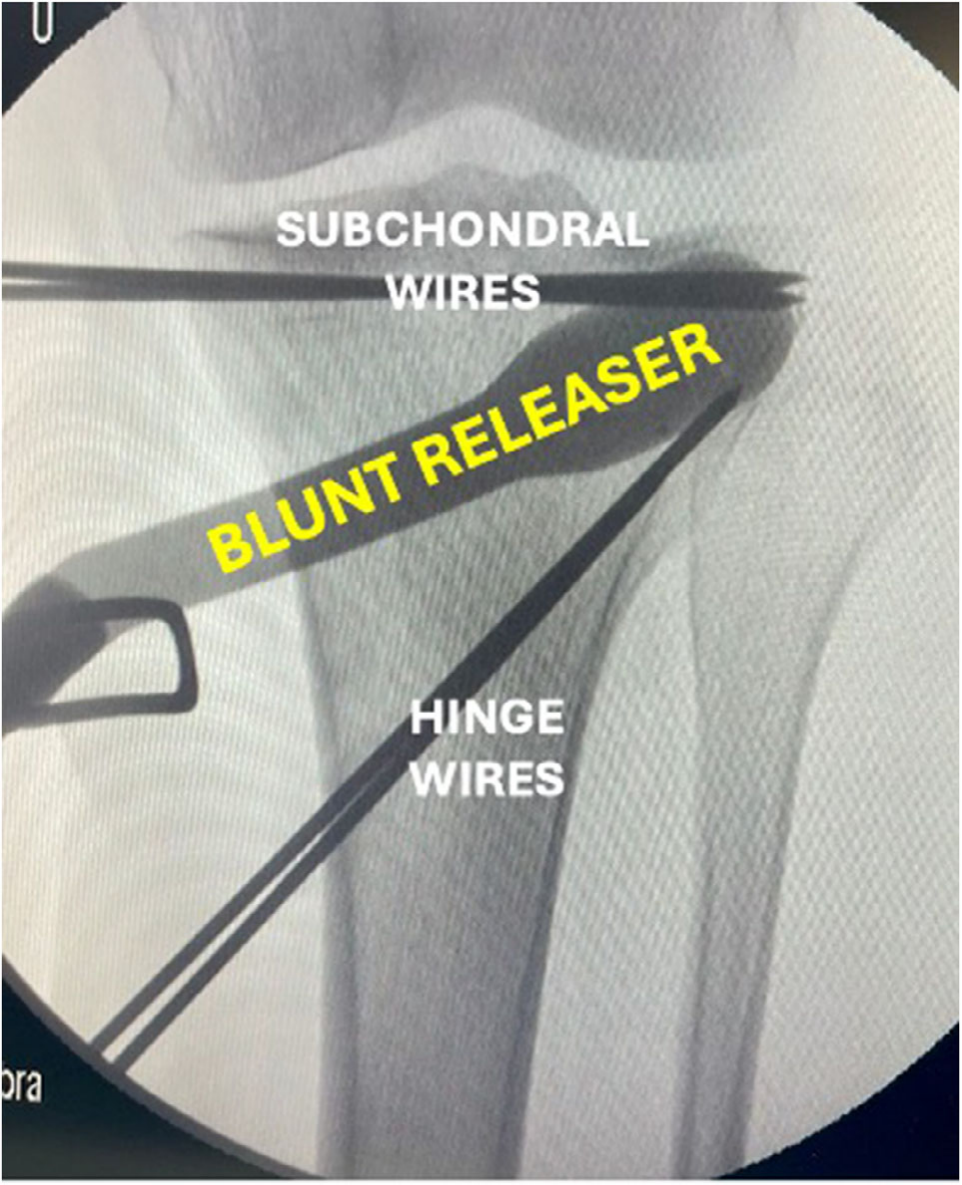
A fluoroscopic view of left proximal tibia showing the blunt releaser creating a safe corridor behind the posterior tibial cortex till the head of fibula.

The releaser is first introduced along the posteromedial tibial wall, staying in constant contact with the bone to ensure posterior neurovascular protection (Figure 3). Once the tip of the releaser reaches the level just above the proximal tibiofibular joint, the protector is slid into the same plane between the posterior cortex and releaser. The releaser is then removed, leaving the protector in situ.

**FIGURE 4:**
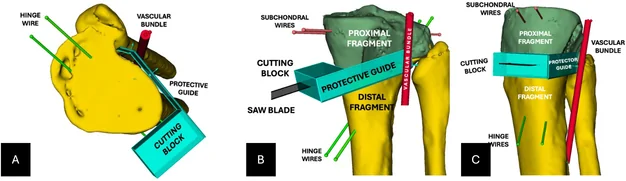
(A) A 3D illustration of right proximal tibia from above after removing the proximal fragment showing the orientation of the hinge wires and contact of the protective guide to posterior cortex of the tibia. (B,C) The protective guide in front of the vascular bundle to allow safe posterior cortical cutting. (3D, 3‐dimensional.)

**FIGURE 5:**
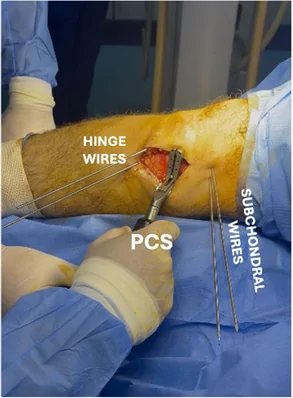
A picture of right proximal tibia after inserting the sentinel wires showing protective cutting system oriented parallel to the native posterior tibial slope before it is fixed to the proximal tibia. (PCS, protective cutting system.)

**FIGURE 6:**
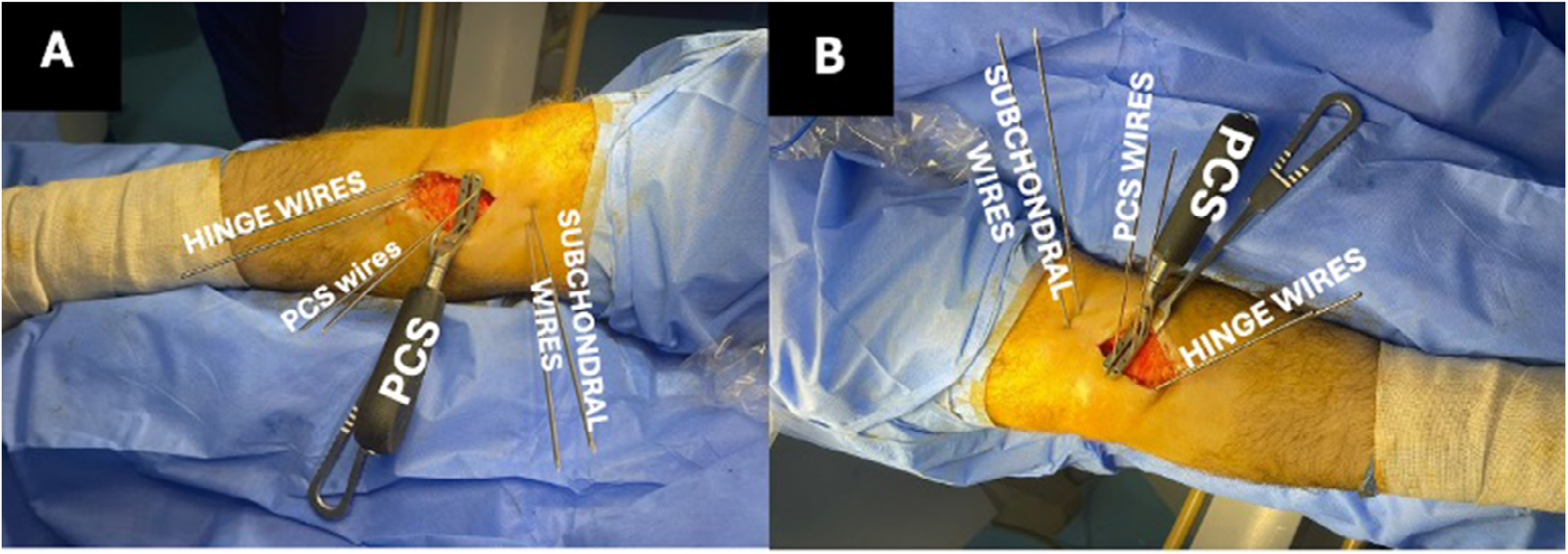
A picture of right proximal tibia after inserting the sentinel wires and PCS wires to fix it to the proximal tibia ((A) medial view and (B) top view). (PCS, protective cutting system.)

**FIGURE 7:**
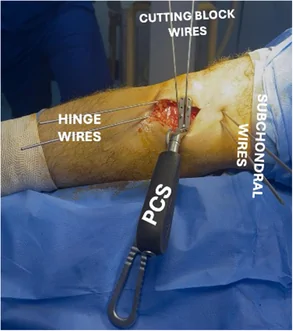
A picture of right proximal tibia after inserting the sentinel wires showing PCS fixed by 2 K‐wires through the slots of the cutting block of PCS. (PCS, protective cutting system.)

Under fluoroscopic control, the tip of the protector is hooked precisely at the planned hinge point, located 10 to 15 mm medial to the lateral tibial cortex and aligned with the upper border of the fibular head. The cutting block is attached to the proximal body of the protector and aligned with the native tibial slope using a lateral fluoroscopic image (Figure 4). The guide pins are inserted through the predrilled holes in the block, fixing it firmly to the medial tibial cortex and defining the saw trajectory (Figures 5, 6, 7).

### Step 3: Osteotomy

**FIGURE 8:**
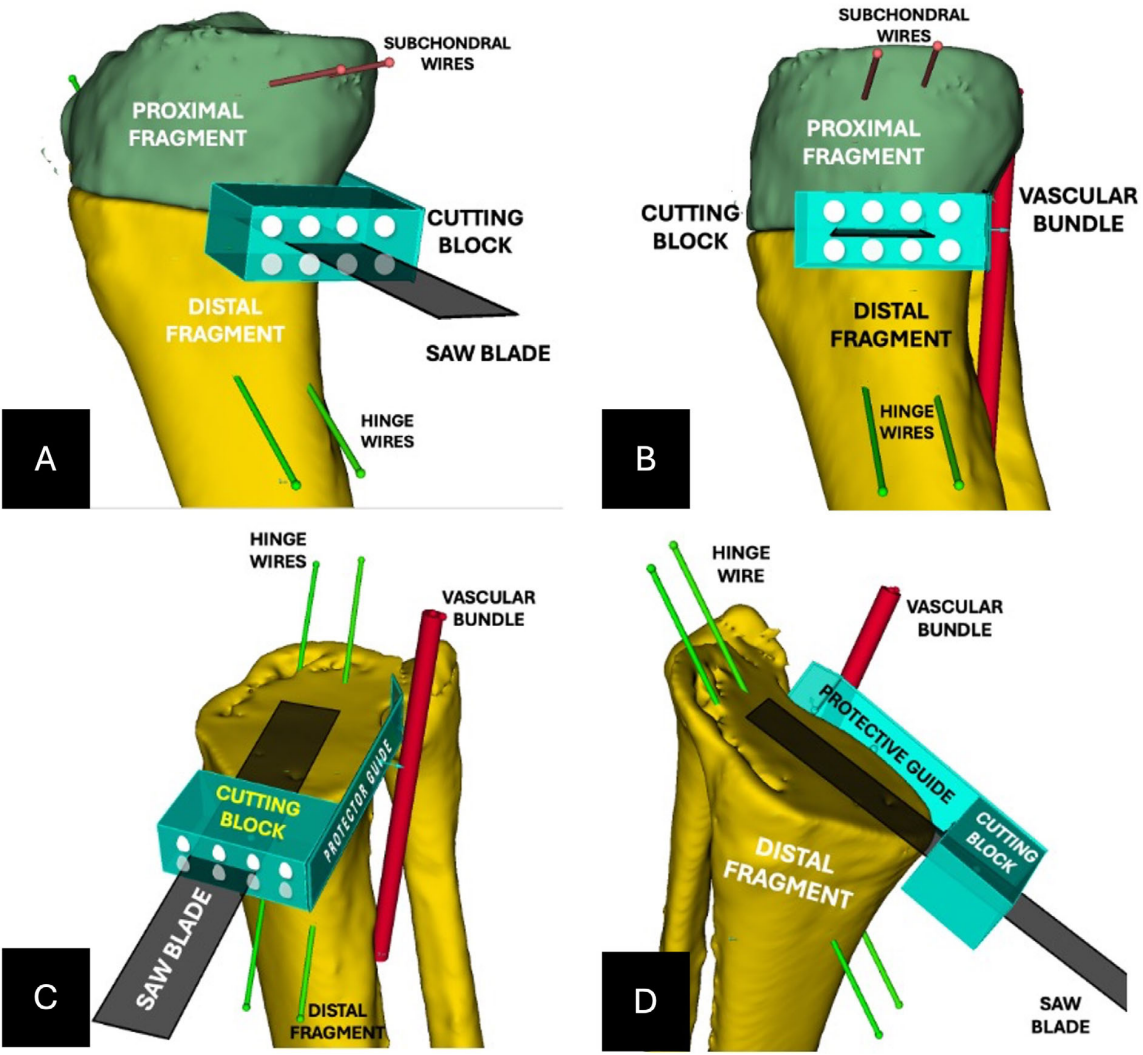
(A) A 3D illustration of right proximal tibia after inserting the sentinel wires showing the position of the PCS over anteromedial tibial surface; (B) the orientation of the slot of the PCS parallel to native tibial slope; (C) a 3D illustration of right proximal tibia after removing the proximal fragment showing how hinge wires can guard against saw progression to lateral tibial cortex; and (D) showing how saw blade motion is controlled by sentinel wires and the protective cutting system. (3D, 3‐dimensional; PCS, protective cutting system.)

Using an oscillating saw blade guided by the central slot of the PCS cutting block, the horizontal osteotomy cut is performed parallel to the slope and directed toward the tip of the protector. The saw movement is physically restricted within the metallic slot of the PCS, preventing posterior advancement beyond the safety limit and ensuring precise cutting along the native tibial slope (Figure 8).

Once the posterior cortex is completely cut—confirmed by gentle contact with the metallic protector tip—the cutting block and protector are withdrawn. The vertical limb of the osteotomy is then made at approximately 110° to the horizontal cut, forming a standard biplanar configuration.

**FIGURE 9:**
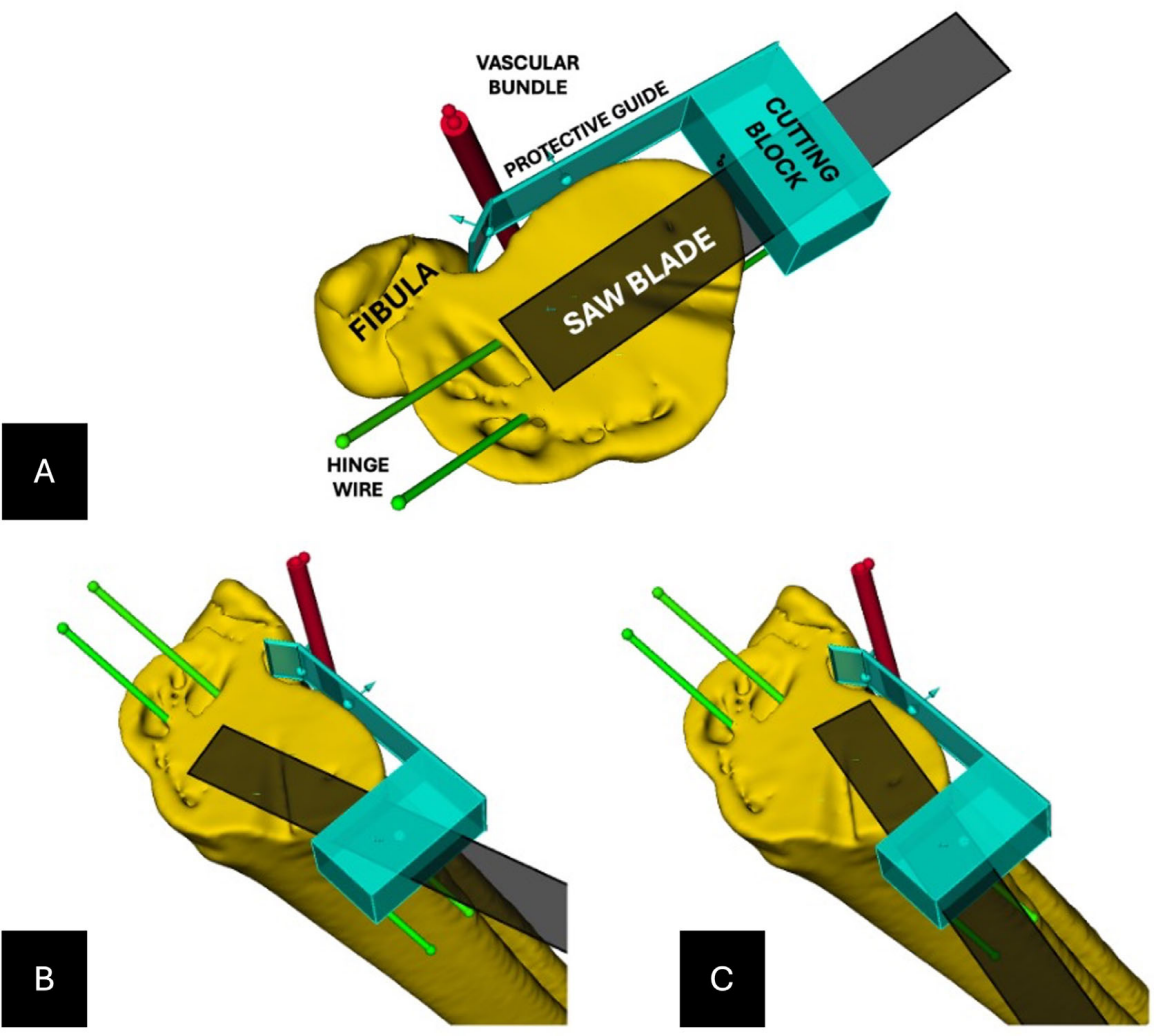
(A) A 3D illustration of right proximal tibia from above after removing the proximal fragment showing how saw blade motion is controlled by sentinel wires and the PCS. (B,C) Showing how saw blade motion is controlled by sentinel wires and the PCS; (B) anterior reflection of lateral cortex is inhibited by 2 hinge wires, and (C) posterior vascular bundle is inhibited through the protective sleeve of the PCS guide. (3D, 3‐dimensional; PCS, protective cutting system.)

In the present technique, 2 hinge wires are used instead of 1, as described in the original sentinel wires study, and function as a mechanical stop. This configuration ensures preservation of at least 10 mm of intact lateral cortex, which is critical for lateral hinge integrity and resistance to hinge fracture (Figures 8 and 9).

### Step 4: Opening the Wedge

**FIGURE 10:**
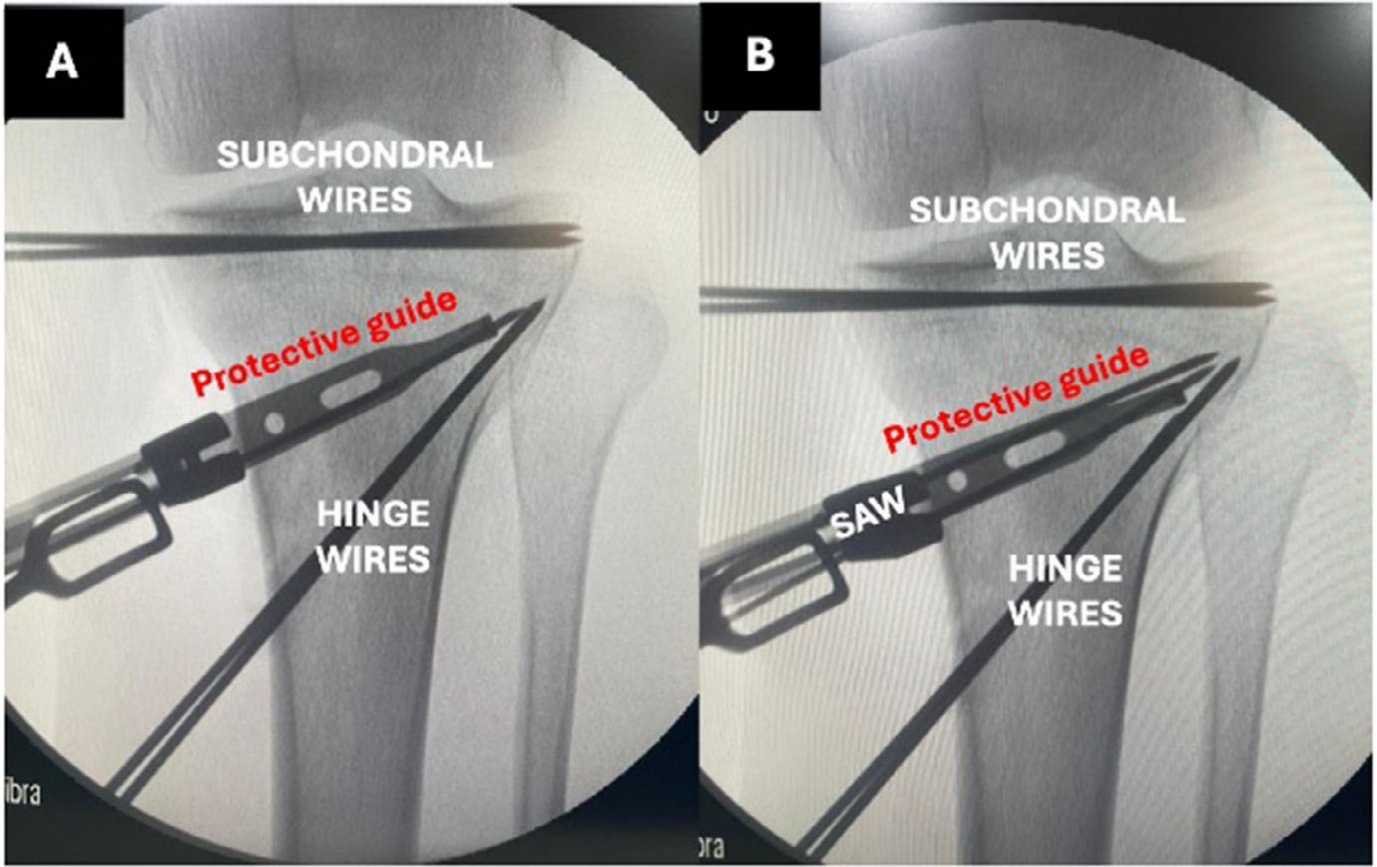
A fluoroscopic view of left proximal tibia showing (A) the position of the protective guide at the planned osteotomy site and (B) how saw blade motion is protected by PCS guide to avoid vascular injury. (PCS, protective cutting system.)

**FIGURE 11:**
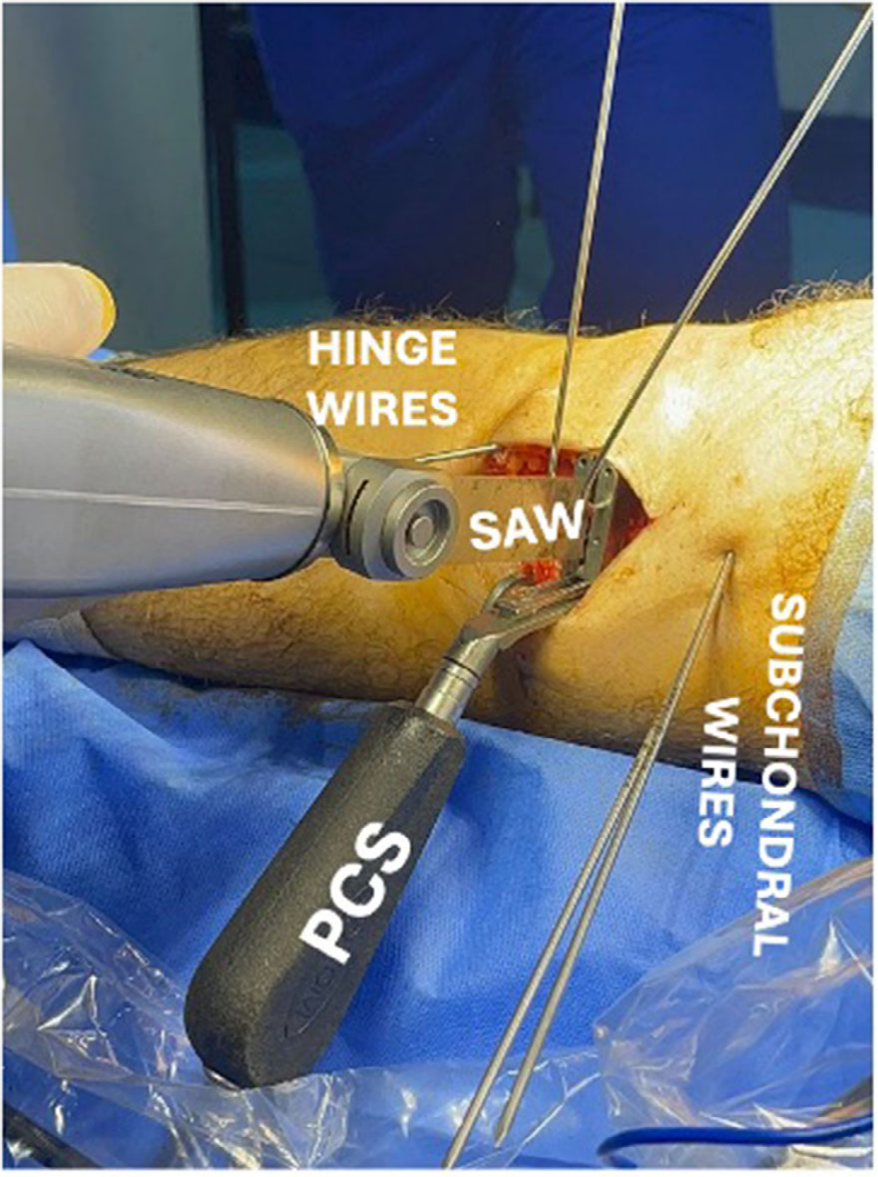
A picture of right proximal tibia after inserting the sentinel wires and the cutting block K‐wires, showing saw blade insertion through the slot of the cutting guide. (PCS, protective cutting system.)

**FIGURE 12:**
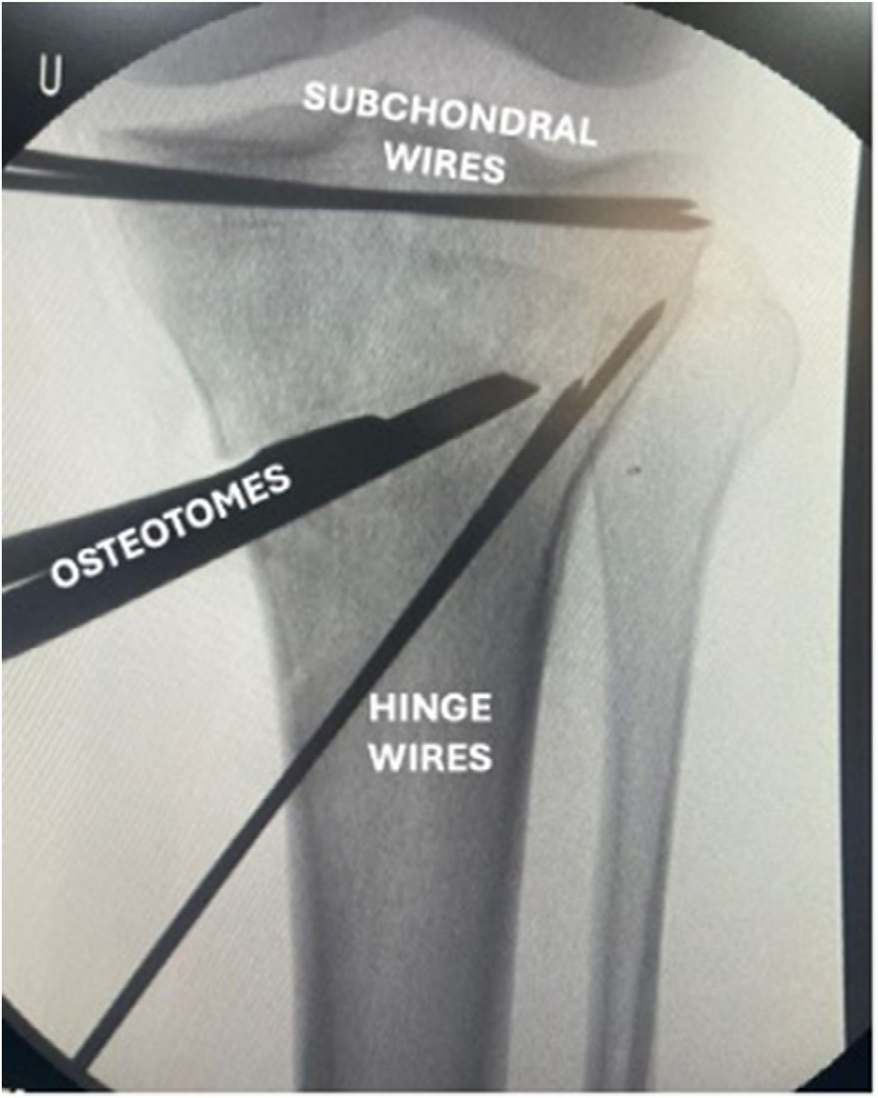
A fluoroscopic view of left proximal tibia showing gradual opening of the osteotomy using multiple osteotomes.

**FIGURE 13:**
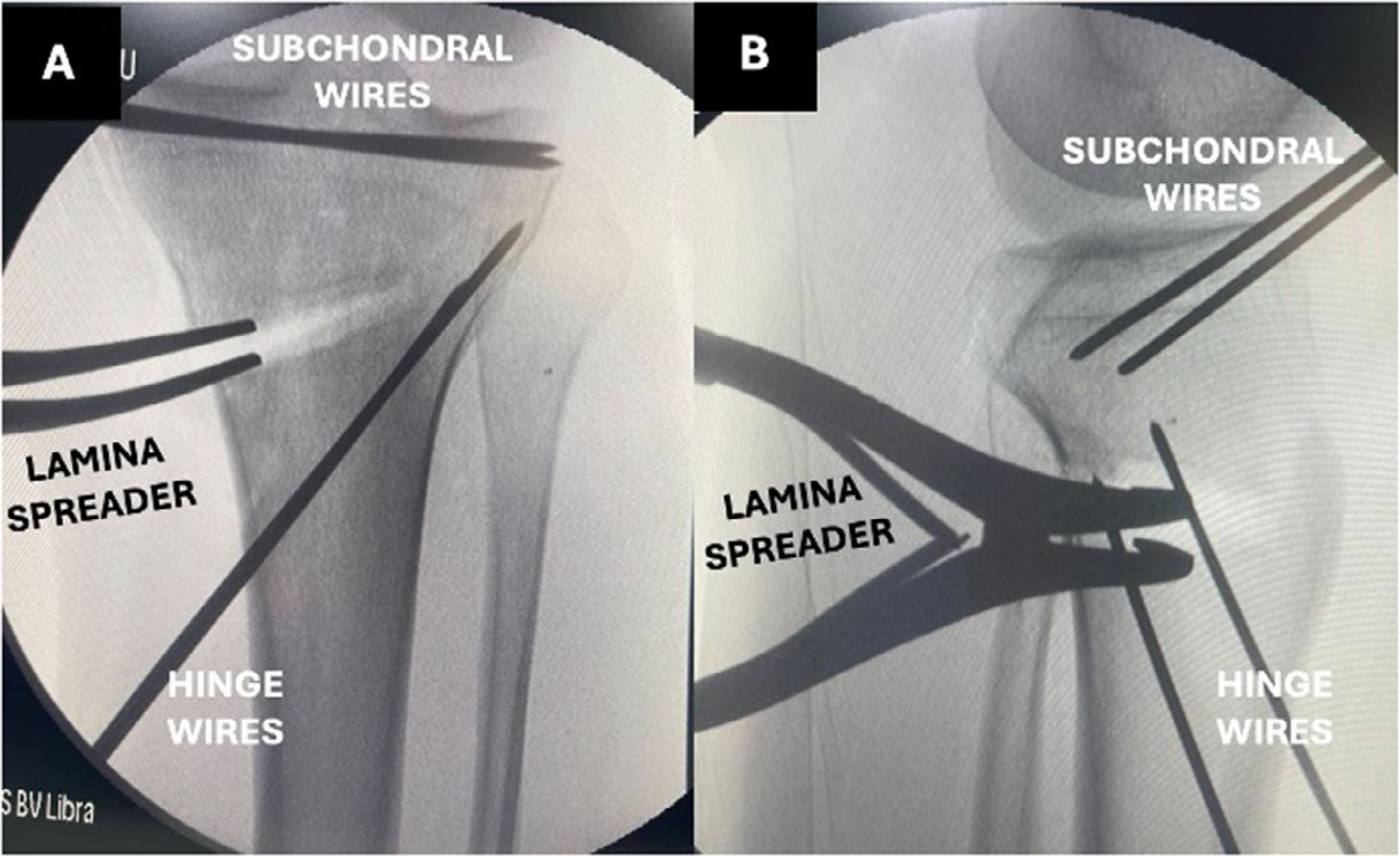
A fluoroscopic view of left proximal tibia ((A) AP and (B) lateral) showing opening of the osteotomy using lamina spreader while hinge and subchondral wires are inserted. (AP, Anteroposterior.)

Progressive osteotomes are inserted sequentially into the osteotomy gap under fluoroscopic control. The posterior lamina spreader is positioned carefully to achieve the desired correction angle without exerting excessive stress on the lateral hinge. During opening, the hinge and subchondral wires remain in place, serving as real‐time mechanical supports against hinge propagation. The opening is continued until the target alignment is achieved, confirmed by a diathermy cable passing through the hip, knee, and ankle centers (Figures 10, 11, 12, 13).

### Step 5: Fixation

**FIGURE 14:**
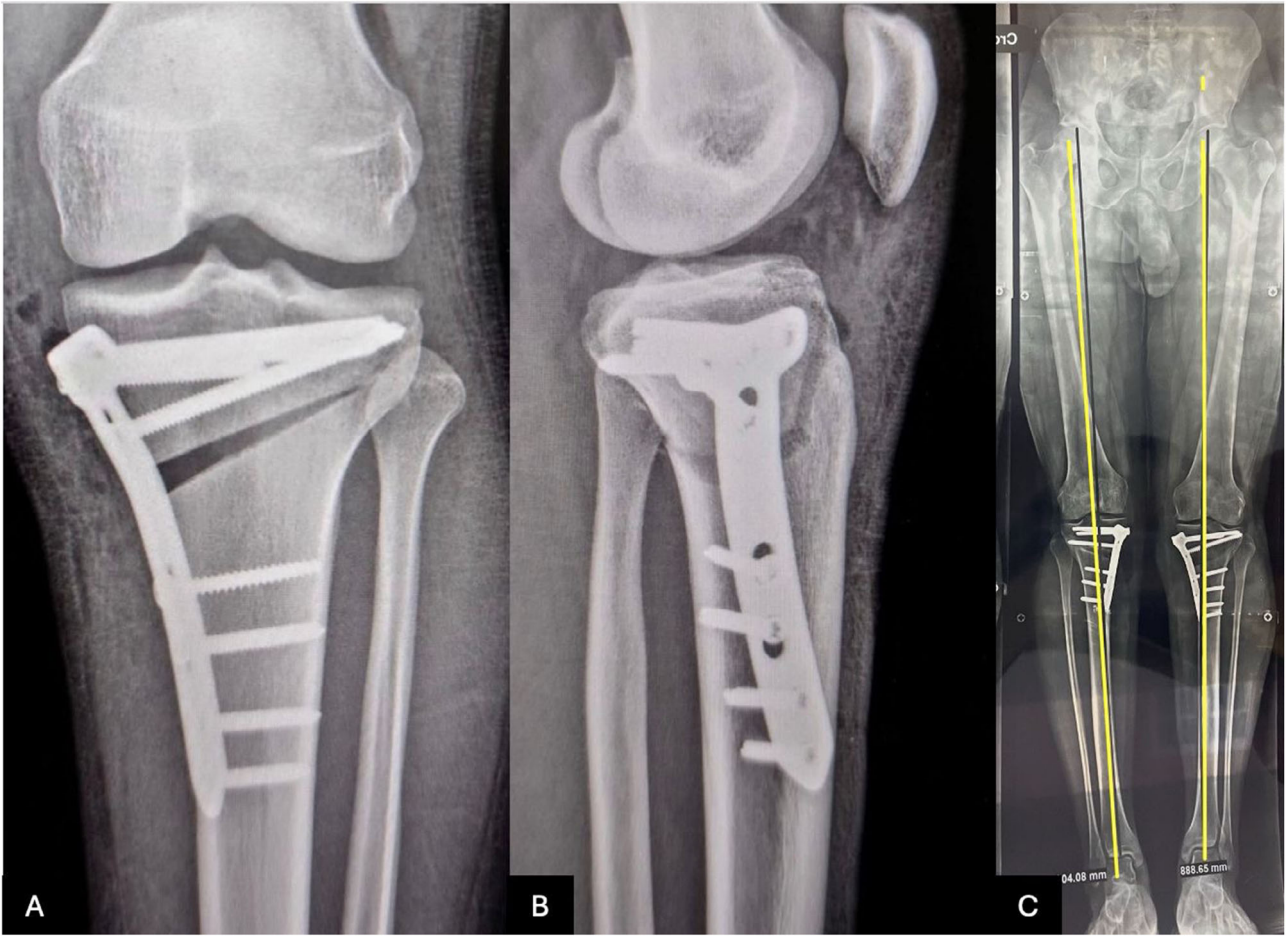
Postoperative radiographs showing medial open‐wedge high tibial osteotomy fixed with a TomoFix plate. (A) Anteroposterior and (B) lateral views of the left knee showing maintained lateral hinge integrity. (C) Full‐length standing anteroposterior radiograph (long‐leg film) of both lower limbs showing restored mechanical axis alignment.

Once the desired correction is achieved, a TomoFix locking plate (DePuy Synthes, Oberdorf, Switzerland) is applied on the medial side. Proximal fixation is performed first, followed by controlled compression of the osteotomy site using a temporary lag screw. This maneuver compresses any induced type 1 hinge microfracture, ensuring stable contact between fragments. Final fixation is achieved with distal locking screws. Bone graft may be inserted within the osteotomy gap if the opening exceeds 10 mm (Figure 14).

### Postoperative Protocol

Postoperatively, partial weight bearing of 15 to 20 kg with crutch assistance is permitted from day 1. Active and passive range‐of‐motion exercises are initiated early and encouraged during the first 4 weeks. Progression to full weight bearing is allowed between weeks 6 and 8 once radiographic evidence of callus formation is confirmed. Routine follow‐up includes standard anteroposterior, lateral, and oblique radiographs to assess hinge integrity and ensure maintenance of the achieved correction.

## DISCUSSION

OWHTO remains a technically demanding procedure, despite its proven efficacy in unloading the medial compartment and delaying knee arthroplasty. The key determinants of a successful outcome are preservation of the lateral cortical hinge, avoidance of posterior neurovascular injury, and maintenance of the native posterior tibial slope. These complications share a common origin: loss of intraoperative control on the tibial cut with regard to the saw direction, cutting depth, and hinge integrity. The HPSS was designed to address these critical limitations by integrating 2 validated safety principles, PCS (Tradimedics, Seongnam, Republic of Korea) and sentinel wires, into a single operative strategy.

The proximity of the popliteal artery and tibial nerve to the posterior tibial cortex places them at risk during osteotomy, especially when cutting posteriorly in varus knees or in large corrections.^4^, ^5^, ^6^, ^7^, ^8^, ^9^, ^10^ Preserving the lateral hinge is crucial for osteotomy stability and bone healing. Takeuchi et al.^3^ classified lateral hinge fractures into 3 types: type 1 (through the osteotomy line, stable), type 2 (distal to tibiofibular joint), and type 3 (through lateral plateau). Among these, type 2 and type 3 are biomechanically unstable and predisposed to loss of correction or delayed union.^11^, ^12^, ^13^, ^14^ Unintended posterior tibial slope increase is a frequent source of postoperative mechanical imbalance and altered cruciate ligament tension.^15^, ^16^, ^17^, ^18^, ^19^, ^20^

The PCS (Tradimedics, Seongnam, Republic of Korea) introduced by Lee et al.^8^ represented a major physical barrier between the oscillating saw and the posterior neurovascular bundle, whereas the cutting slot ensured a uniplanar, slope‐aligned osteotomy. By maintaining continuous bone contact, the PCS (Tradimedics, Seongnam, Republic of Korea) not only prevented inadvertent posterior penetration but also allowed precise control of the osteotomy level and slope. However, the PCS does not reinforce the lateral cortex mechanically. The lateral hinge remains vulnerable to propagation fractures (Takeuchi type 2 and 3) during wedge opening, particularly in large corrections or osteoporotic bone. The sentinel wires technique, described by Gawish et al.,^9^ focused on hinge stability through a strategic network of K‐wires. Yet, because the osteotomy is performed freehand, the posterior neurovascular structures remain exposed, and the saw movement lacks physical limitation beyond fluoroscopic guidance. The PCS (Tradimedics, Seongnam, Republic of Korea) component of HPSS allows direct intraoperative alignment of the saw with the native slope using the superior surface of the cutting block under fluoroscopic guidance. Because the block is fixed with guide pins, slope orientation remains stable during cutting. The sentinel wire construct further maintains hinge position during wedge opening, ensuring symmetric expansion of the osteotomy gap anteriorly and posteriorly. This synergy provides precise correction control and minimizes slope alterations—a combination rarely achievable with freehand methods. In conclusion, the HPSS could provide a reliable solution to the main technical challenges of OWHTO by simultaneously protecting posterior neurovascular structures, preserving the lateral cortical hinge, and maintaining the native posterior tibial slope. By integrating guided slope‐aligned cutting with mechanical hinge reinforcement, HPSS enhances intraoperative control and reduces the risk of major complications, offering a safe and reproducible technique for OWHTO.

## DISCLOSURES

The authors (A.E., M.D., I.M., M.G., B.A.E., H.G.) declare that they have no known competing financial interests or personal relationships that could have appeared to influence the work reported in this article.
